# Network-driven prioritization and functional phenotyping nominate TTC23 as a biomarker-informed target in chlorpromazine repurposing for glioblastoma

**DOI:** 10.3389/fphar.2026.1797067

**Published:** 2026-05-04

**Authors:** Jianqiang Hao, Hongbin Liu, Yongqiang Ye, Jianwei Lv, Fang Xue, Yanli Cai

**Affiliations:** 1 Department of Neurosurgery, Ziyang Central Hospital, West China Hospital of Sichuan University-Ziyang Hospital, Ziyang, China; 2 Outpatient Department, Ziyang Central Hospital, West China Hospital of Sichuan University-Ziyang Hospital, Ziyang, China

**Keywords:** chlorpromazine, drug repurposing, glioblastoma, network pharmacology, prognostic modeling, TTC23, tumor immunity

## Abstract

**Background:**

Glioblastoma (GBM) remains a lethal brain tumor with limited therapeutic options and near-universal recurrence. Drug repurposing offers a practical strategy, but pleiotropic compounds require systematic target triage to yield actionable and testable vulnerabilities.

**Methods:**

We integrated GBM transcriptomic dysregulation with curated chlorpromazine (CPZ)-associated targets to define drug–disease intersecting genes, constructed a protein–protein interaction network, and developed an outcome-linked Lasso–Cox prognostic model to prioritize core candidates. Structure-informed docking and coarse-grained conformational sampling were used to evaluate the plausibility of a TTC23–CPZ interaction. TTC23-associated pathway activity, oncogenic state features, and immune contexture were characterized using expression stratification, enrichment and state scoring, cancer–immunity cycle analysis, and immune infiltration estimation. Functional validation was performed in GBM cell models to assess migration, apoptosis, cell viability, and clonogenic potential under TTC23 perturbation with or without CPZ exposure.

**Results:**

Integrated CPZ–GBM intersection analysis and network-based prognostic modeling consistently prioritized TTC23 as a clinically relevant candidate. Structure-based analyses supported a consistent TTC23–CPZ interaction hypothesis across conformational sampling. Elevated TTC23 expression was associated with coordinated pathway activation, malignant functional states, and distinct immune-associated features. Functionally, TTC23 depletion suppressed migratory capacity, increased apoptotic susceptibility, reduced short-term viability, and impaired long-term clonogenic survival, while sensitizing GBM cells to CPZ-associated anti-tumor phenotypes.

**Conclusion:**

Our multi-layer framework nominates TTC23 as a functionally relevant determinant associated with CPZ response in GBM and supports the CPZ–TTC23 axis as a candidate for biomarker-informed drug repurposing.

## 1 Introduction

Primary malignant tumors of the brain and central nervous system (CNS) impose a disproportionate health burden because of their high lethality and profound neurological morbidity (Kim et al., 2025a). In 2022, brain/CNS cancers were estimated to account for ∼321,624 new cases and ∼248,403 deaths worldwide, reflecting a persistently poor global outcome despite incremental advances in neuro-oncology (Bray et al., 2024). In the United States, glioblastoma represents the archetypal aggressive entity within malignant CNS tumors: contemporary population-based data show a 5 year relative survival of ∼7.0% for glioblastoma across all ages (Ostrom et al., 2023; Li et al., 2025), underscoring the urgent need for tractable therapeutic vulnerabilities that can translate into durable disease control.

Current first-line management for newly diagnosed glioblastoma remains maximal safe resection followed by radiotherapy with concomitant and adjuvant temozolomide (the Stupp regimen) (Davis, 2016; Oronsky et al., 2020; Xiong et al., 2024). However, the clinical benefit remains limited: in the pivotal trial that established this standard, median overall survival improved only modestly (approximately 14.6 months vs. 12.1 months with radiotherapy alone), and recurrence remains the rule rather than the exception (Stupp et al., 2005). Multiple biological and therapeutic barriers converge to constrain progress, including profound intratumoral heterogeneity, rapid adaptive rewiring under treatment pressure, and the pharmacologic challenge of achieving effective intratumoral exposure across the blood–brain barrier (BBB) (García-Montaño et al., 2023; Ahmed et al., 2023; Wen et al., 2025). Together, these constraints motivate complementary strategies that (i) leverage BBB-penetrant compounds, (ii) exploit polypharmacology to match pathway redundancy, and (iii) identify biomarkers that can prioritize patients and mechanistically anchor drug responses.

Drug repurposing provides a pragmatic route to accelerate such efforts because clinically used molecules come with known pharmacokinetics and safety frameworks (Lyne and Yamini, 2021; Alomari et al., 2021). Chlorpromazine (CPZ), a lipophilic phenothiazine antipsychotic with established CNS distribution, has attracted renewed interest as a potential anti-glioblastoma agent (Abbruzzese et al., 2020; Abbruzzese et al., 2023). Preclinical studies indicate that CPZ can perturb stress-adaptation programs relevant to glioblastoma cell survival, including ER stress/UPR-linked cytotoxic autophagy and lysosome-related vulnerabilities (Matteoni et al., 2021; Jacob et al., 2024). Importantly, early clinical exploration has suggested feasibility of adding CPZ to temozolomide-based regimens in selected settings (e.g., the RACTAC phase II experience) while broader prospective evaluation continues (e.g., registered studies assessing CPZ within standard-of-care protocols) (Pace et al., 2023). Nevertheless, a key gap remains: for CPZ to be advanced rationally in glioblastoma, its candidate target landscape and the tumor-context dependencies that determine response need to be clarified, ideally in a way that links molecular networks to clinically meaningful risk stratification.

In this study, we address this gap by integrating transcriptomic dysregulation in glioblastoma with curated CPZ-associated targets to define a CPZ-relevant disease module, rather than presuming single-pathway dependence. We then translate this module into a prognostic framework by constructing a penalized Cox model to derive a clinically informative risk score, and we prioritize TTC23 as a core candidate based on its consistent performance across network centrality metrics, survival modeling, and downstream validation feasibility. To strengthen target plausibility beyond correlative association, we further apply structure-based modeling to evaluate the structural compatibility and conformational stability of a putative TTC23–CPZ interaction. Finally, we interpret TTC23-associated biology using pathway-activity and tumor-immunity analyses to connect CPZ-relevant networks with functional states and immune contexture, providing a structured rationale for subsequent experimental evaluation. Collectively, this multi-layered framework aims to position CPZ repurposing for glioblastoma within an interpretable, biomarker-informed hypothesis rather than to assert immediate clinical translatability.

## 2 Methods

### 2.1 Transcriptomic data processing and target integration

Gene expression data for glioblastoma (GBM) were obtained from the Gene Expression Omnibus (GEO) under accession GSE4290 and used as the discovery cohort for comparisons between tumor and non-tumor brain tissues (Clough and Barrett, 2016). Samples were classified according to GEO annotations. Gene identifiers were unified to official HGNC symbols, and duplicated symbols were collapsed to a single representative expression value. Putative molecular targets of chlorpromazine (CPZ) were compiled from multiple public resources, including the Comparative Toxicogenomics Database (CTD), Similarity Ensemble Approach (SEA) (Keiser et al., 2009), STITCH, SwissTargetPrediction (Daina et al., 2019), and PubChem (Kim et al., 2025b). After gene symbol standardization and deduplication, a non-redundant CPZ target set was obtained for downstream integration analyses.

Differential expression analysis between GBM and non-tumor samples was performed at the gene level, with multiple testing controlled using the Benjamini–Hochberg false discovery rate procedure. Genes meeting adjusted P < 0.05 and |log2 fold change| > 0.5 were defined as differentially expressed genes (DEGs). For intersection analyses, DEGs were further stratified into upregulated and downregulated subsets using more stringent fold-change thresholds. Overlap between CPZ targets and GBM DEG subsets was summarized using an UpSet-style visualization to delineate intersecting candidate genes (Conway et al., 2017).

### 2.2 Network construction and prognostic modeling

Protein–protein interaction (PPI) analysis was conducted for the intersecting gene set using the STRING database (Szklarczyk et al., 2023), retaining only high-confidence interactions. The resulting interaction network was visualized in Cytoscape (Shannon et al., 2003), and overall network topology was examined to characterize connectivity patterns among CPZ-associated and GBM-dysregulated genes. To evaluate the clinical relevance of the intersecting gene set, Lasso-penalized Cox proportional hazards regression was applied to construct a parsimonious prognostic model (Tibshirani, 1997; Simon et al., 2011). Ten-fold cross-validation was used to select the optimal regularization parameter, and genes with non-zero coefficients were retained in the final signature. A composite risk score was calculated for each patient as the weighted sum of gene expression values and corresponding coefficients, and patients were stratified into high- and low-risk groups using the median score as the cutoff. Survival differences were assessed using Kaplan-Meier analysis with the log-rank test. To summarize prognostic performance across clinical endpoints, hazard ratios for overall survival, disease-specific survival, and progression-free interval were pooled using a random-effects meta-analysis framework. Between-study heterogeneity was evaluated using I^2^ and τ^2^ statistics, and results were presented as forest plots (DerSimonian and Laird, 1986; Higgins and Thompson, 2002).

### 2.3 Functional landscape analysis of CPZ–GBM intersecting targets

To characterize the global biological context represented by CPZ–GBM intersecting genes, pathway enrichment analysis was performed using the KEGG reference database (Kanehisa et al., 2025). Enriched pathways and representative genes were visualized in a drug–target–pathway Sankey diagram to illustrate major pharmacological linkages. Gene Ontology enrichment analyses were conducted to annotate biological processes (Ashburner et al., 2000), cellular components, and molecular functions associated with the intersecting gene set, and dominant functional themes were summarized using a circular visualization. Enrichment results passing multiple-testing adjustment were retained for interpretation as a systems-level functional backdrop.

### 2.4 Structure-based modeling and conformational stability assessment

Structure-based analyses were performed to evaluate the plausibility of chlorpromazine (CPZ) interacting with TTC23. Molecular docking was conducted using a three-dimensional structural model of TTC23 and an optimized CPZ ligand, following standard receptor and ligand preparation procedures. Docking poses were ranked by scoring metrics and interaction plausibility, and the top-ranked representative pose was selected for visualization and analysis of electrostatic surface features and pocket geometry.

To assess conformational behavior beyond static docking, coarse-grained conformational sampling was performed using CABS-flex based on the docked complex (Kuriata et al., 2018). Structural ensembles were analyzed by superposition to evaluate global flexibility, residue contact frequency maps to assess interaction persistence, and per-residue root mean square fluctuation (RMSF) profiles to quantify local mobility. Relatively low fluctuations observed within the predicted ligand-binding pocket were interpreted as supporting the structural plausibility of a putative TTC23–CPZ interaction under conformational sampling, rather than as definitive evidence of binding.

### 2.5 Expression profiling and diagnostic evaluation in bulk transcriptomic cohorts

TTC23 expression in glioblastoma was first evaluated using RNA-sequencing data from the TCGA-GBM cohort (Cancer Genome Atlas Research Networks, 2008). To assess tumor specificity, normal brain samples from the GTEx project were used as a reference and compared with TCGA-GBM samples using harmonized expression units and consistent gene-symbol mapping, with results interpreted cautiously in light of platform and cohort differences (GTEx Consortium, 2020). Diagnostic performance was evaluated using receiver operating characteristic (ROC) curve analysis (Robin et al., 2011). Associations between TTC23 expression and patient age were assessed using correlation analysis to evaluate potential confounding effects.

### 2.6 Single-cell cell-cycle–resolved expression analysis

Cell-cycle–dependent expression of TTC23 was examined using the GSE146773 dataset, which comprises 1,152 single U2OS FUCCI cells sorted by fluorescence-activated cell sorting and profiled by SMART-seq2–based single-cell RNA sequencing (Mahdessian et al., 2021; Picelli et al., 2014). Cell-cycle phases were determined based on FUCCI fluorescence intensities, enabling assignment of individual cells to defined cell-cycle states and ordering along a cell-cycle pseudotime axis. Gene expression values were scaled to z-scores, and extreme outliers (z > 3 or z < −3) were excluded. Differences in TTC23 expression across cell-cycle phases were evaluated using the Kruskal–Wallis rank-sum test. Dynamic expression trends along cell-cycle pseudotime were visualized to assess continuous changes associated with cell-cycle progression (Sakaue-Sawano et al., 2008).

### 2.7 Pathway enrichment and oncogenic state analysis based on TTC23 expression

Samples were stratified into TTC23 high- and low-expression groups by defining the top 30% and bottom 30% of samples according to TTC23 transcript levels. Differential expression analysis between the two groups was performed using the limma framework (Ritchie et al., 2015), and genes were ranked by log2 fold change or ΔZ-score to generate ordered gene lists. Gene set enrichment analysis (GSEA) (Subramanian et al., 2005) was conducted using the clusterProfiler package (Yu et al., 2012), with KEGG and Hallmark gene sets as references, and normalized enrichment scores (NES) were calculated. Statistical significance was evaluated using permutation-based testing followed by multiple hypothesis correction. In parallel, tumor functional states were characterized using 14 oncogenic phenotype signatures curated from the CancerSEA resource. Pathway activity scores were calculated using the GSVA package (Hänzelmann et al., 2013) with the z-score method and subsequently standardized using the scale function to obtain comparable gene set scores. Pearson correlation analysis was performed to assess associations between TTC23 expression and oncogenic state scores, enabling identification of selectively enriched functional programs linked to TTC23 dysregulation.

### 2.8 Cancer–immunity cycle activity and immunomodulator regulation analysis

Anti-cancer immune activity was systematically evaluated using the Tracking Tumor Immunophenotype (TIP) framework (Xu et al., 2018), which integrates ssGSEA- and CIBERSORT-based approaches to quantify immune activity across the seven-step cancer–immunity cycle, including antigen release, antigen presentation, immune activation, immune cell trafficking, tumor infiltration, cancer cell recognition, and cancer cell killing (Barbie et al., 2009; Newman et al., 2015). TIP scores were calculated for each tumor sample, and Spearman correlation analysis was used to assess associations between TTC23 expression and individual immune cycle steps, as well as inter-step correlations. Visualization was performed using the linkET package. To further characterize immune regulatory patterns, immunostimulatory genes, immunoinhibitory genes, chemokines, and human leukocyte antigen (HLA) genes were retrieved from the TISIDB database (Ru et al., 2019). Tumor samples were stratified into TTC23 high- and low-expression groups, and differential expression of immune-related genes was assessed using the Wilcoxon rank-sum test, with mean expression values visualized as heatmaps.

### 2.9 Immune regulatory architecture and immune infiltration profiling

To investigate integrated immune regulatory mechanisms, immunomodulators were further analyzed across TTC23 expression quartiles (Q1–Q4). For each subgroup, median mRNA expression levels, expression–DNA methylation correlations, and somatic copy number alteration (SCNA) frequencies, including amplification and deletion events, were calculated and visualized using heatmap-based representations following established immune landscape frameworks. In addition, immune response signatures and genome instability–related scores were evaluated across TTC23 expression quartiles, with subgroup-wise averages computed and row-wise standardization applied to enable cross-feature comparison. Immune cell infiltration was estimated using two complementary approaches. First, ssGSEA-based infiltration scores were calculated using the GSVA package with curated marker genes for 24 immune cell types, followed by Spearman correlation analysis with TTC23 expression. Second, immune cell proportions were estimated using the CIBERSORT algorithm with reference immune cell signatures. Group-wise infiltration patterns were visualized using lollipop plots and stacked bar charts to illustrate TTC23-associated immune landscape alterations.

### 2.10 Cell lines and cell culture

Human glioblastoma cell lines U87MG and U251MG, as well as the human embryonic kidney cell line HEK293T, were used in this study. U87MG (RRID: CVCL_0022), U251MG (RRID: CVCL_0021), and HEK293T (RRID: CVCL_0063) were obtained from the American Type Culture Collection (ATCC). U87MG and U251MG cells were cultured in Dulbecco’s Modified Eagle’s Medium (DMEM; high glucose) supplemented with 10% fetal bovine serum (FBS) and 1% penicillin–streptomycin. HEK293T cells were maintained under the same culture conditions. All cells were incubated at 37 °C in a humidified atmosphere containing 5% CO_2_ and routinely passaged at 70%–80% confluence. All cell lines were routinely tested and confirmed to be free of *mycoplasma* contamination.

### 2.11 Construction of stable shTTC23 knockdown cell lines

Stable TTC23 knockdown cell lines were generated using a lentiviral shRNA system. Short hairpin RNAs targeting TTC23 (shTTC23) and a non-targeting control shRNA (shNC) were cloned into lentiviral vectors. Lentiviruses were packaged in HEK293T cells and subsequently used to infect U87MG and U251MG cells. After infection, cells were subjected to antibiotic selection to establish stable knockdown cell lines. Knockdown efficiency was validated at the mRNA level prior to subsequent functional assays.

### 2.12 Quantitative real-time PCR

Total RNA was extracted from cells using TRIzol reagent according to the manufacturer’s instructions. RNA concentration and purity were assessed spectrophotometrically. Complementary DNA (cDNA) was synthesized from total RNA using a reverse transcription kit. Quantitative real-time PCR was performed using SYBR Green–based chemistry on a real-time PCR system. Gene-specific primers were used for amplification, and glyceraldehyde-3-phosphate dehydrogenase (GAPDH) was used as an internal control. Relative mRNA expression levels were calculated using the 2^−ΔΔCt^ method. All reactions were performed in triplicate, and each experiment was independently repeated at least three times. The primer sequences used in this study were as follows:

GAPDH-F: GAC​AGT​CAG​CCG​CAT​CTT​CT.
GAPDH-R: GCG​CCC​AAT​ACG​ACC​AAA​TC.
TTC23-F: GCA​GGA​AGA​AGA​GTG​TTC​AGA​GA.
TTC23-R: GGT​CCC​ACA​AAC​CAT​CCT​CT.
MMP9-F: TCT​ATG​GTC​CTC​GCC​CTG​AA.
MMP9-R: CAT​CGT​CCA​CCG​GAC​TCA​AA.
CDH1-F: AAA​ACA​GCA​AAG​GGC​TTG​GAT​T.
CDH1-R: AGC​CAG​TTG​GCA​GTG​TCT​CT.
CXCL10-F: GCT​TCC​AAG​GAT​GGA​CCA​CA.
CXCL10-R: GCA​GGG​TCA​GAA​CAT​CCA​CT.
CCL2-F: AGA​GGC​TGA​GAC​TAA​CCC​AGA.
CCL2-R: TTT​CAT​GCT​GGA​GGC​GAG​AG.
CD274-F: TGC​AGG​GCA​TTC​CAG​AAA​GA.
CD274-R: ATG​CGT​TCA​GCA​AAT​GCC​AG.

### 2.13 Cell colony formation

For the colony formation assay, U87MG and U251MG cells were seeded into six-well plates at a low density. Assay-specific CPZ concentrations were selected based on preliminary dose–response optimization to maintain an appropriate dynamic range for each readout while avoiding excessive non-specific cytotoxicity. After cell attachment, cells were treated with chlorpromazine (CPZ) at a final concentration of 1 μM as indicated, while control cells received an equal volume of vehicle. Cells were maintained under standard culture conditions, and the medium was replaced at regular intervals. After an appropriate incubation period to allow colony formation, colonies were fixed and stained. Colonies containing more than 50 cells were counted for quantitative analysis.

### 2.14 Cell viability assay (CCK-8)

Cell viability was evaluated using the Cell Counting Kit-8 (CCK-8; Dojindo Laboratories, Kumamoto, Japan; Cat. No. CK04). U87MG and U251MG cells were seeded into 96-well plates and allowed to attach overnight. Cells were then treated with chlorpromazine (CPZ) at a final concentration of 5 μM as indicated, while control groups received an equal volume of vehicle. Cell viability was assessed at 0, 24, 48, 72, and 96 h after treatment. At each time point, CCK-8 reagent was added to each well and incubated at 37 °C protected from light. Absorbance was measured at 450 nm using a microplate reader. Background absorbance was subtracted, and cell viability was normalized to the corresponding control group. Each condition was tested in technical replicates, and all experiments were independently repeated at least three times.

### 2.15 Wound healing assay

Cell migratory capacity was evaluated using a wound healing assay. U87MG cells were seeded into six-well plates and cultured until reaching approximately 90% confluence. A uniform linear wound was generated by scratching the cell monolayer with a sterile pipette tip. Detached cells were gently removed by washing with phosphate-buffered saline, and fresh culture medium was added. Cells were then treated under the indicated conditions (shNC, shTTC23, shNC + CPZ, and shTTC23 + CPZ). For CPZ-treated groups, chlorpromazine (CPZ) was applied at a final concentration of 5 μM. Images of the wound areas were captured immediately after scratching (0 h) and after 48 h using an inverted microscope. Wound closure was quantified using image analysis software, and the migration rate was calculated based on the relative reduction in wound width compared with the initial wound. Each experiment was performed in triplicate and independently repeated at least three times.

### 2.16 Annexin V/PI apoptosis assay

Cell apoptosis was analyzed using an Annexin V–FITC/propidium iodide (PI) apoptosis detection kit (BD Biosciences, CA, United States of America; Cat. No. 556547). U87MG and U251MG cells were seeded into six-well plates and treated under the indicated conditions (shNC, shTTC23, shNC + CPZ, and shTTC23 + CPZ). For CPZ-treated groups, chlorpromazine (CPZ) was applied at a final concentration of 10 μM. After treatment for 72 h, cells were collected, washed with cold phosphate-buffered saline, and resuspended in binding buffer. Cells were then stained with Annexin V–FITC and PI according to the manufacturer’s instructions and analyzed by flow cytometry. The percentages of apoptotic cells were quantified, and data analysis was performed using flow cytometry analysis software. Each experiment was conducted in triplicate and independently repeated at least three times.

### 2.17 Statistical analysis

All statistical analyses were performed using R. Continuous variables are presented as mean ± standard deviation (SD) unless otherwise specified. For comparisons between two groups, an unpaired two-tailed Student’s t-test was used for normally distributed data, and the Wilcoxon rank-sum test was applied when normality assumptions were not met. Comparisons among multiple groups were conducted using one-way analysis of variance (ANOVA) with appropriate post hoc tests or the Kruskal–Wallis test, as indicated. For time-course experiments, including cell viability and clonogenic assays, two-way ANOVA was applied to evaluate group, time, and interaction effects. Correlation analyses were performed using Pearson or Spearman methods according to data distribution. Survival differences were assessed using Kaplan-Meier analysis with the log-rank test, and hazard ratios were estimated using Cox proportional hazards models. For transcriptomic and pathway enrichment analyses, multiple testing was controlled using the Benjamini–Hochberg false discovery rate (FDR) procedure unless otherwise stated. A two-sided P value <0.05 was considered statistically significant.

## 3 Results

### 3.1 Integrated transcriptomic screening and prognostic prioritization of CPZ-associated candidates in GBM

**FIGURE 1:**
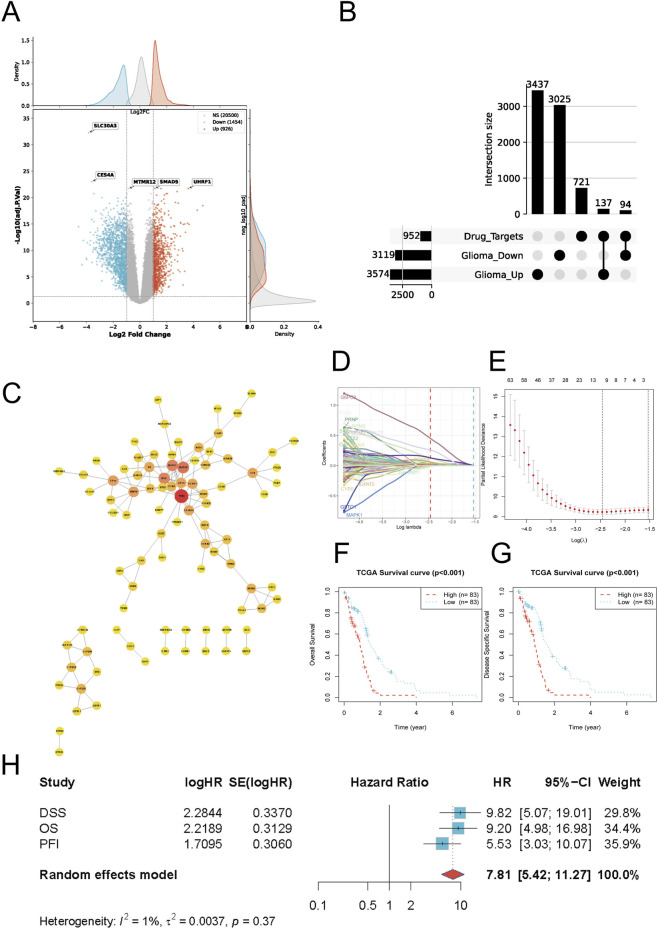
Systematic screening and prognostic modeling. **(A)** Volcano plot of differential expression between GBM and non-tumor brain tissues in GSE4290. Significance is defined by adjusted P-value and log2 fold-change thresholds; counts of upregulated, downregulated, and non-significant genes are shown. **(B)** UpSet intersection analysis among predicted chlorpromazine (CPZ) targets and GBM DEGs stratified as Glioma_Up and Glioma_Down. Bars indicate intersection sizes; left bars indicate set sizes. **(C)** Protein–protein interaction (PPI) network of intersecting genes constructed from STRING high-confidence interactions and visualized in Cytoscape. **(D)** Lasso coefficient profiles across log(λ), showing shrinkage and retention of prognostic features as penalization increases. **(E)** Ten-fold cross-validation curve for Lasso–Cox regression; dashed vertical lines indicate the selected λ. **(F,G)** Kaplan–Meier survival curves of the risk model in TCGA, comparing high-vs. low-risk groups for overall survival (OS) **(F)** and disease-specific survival (DSS) **(G)**. **(H)** Forest-plot summary of hazard ratios (HRs) across OS, DSS, and progression-free interval (PFI).

We first characterized transcriptomic alterations in glioblastoma (GBM) using the GSE4290 dataset, identifying widespread dysregulation between tumor and non-tumor brain tissues, including 926 upregulated and 1,454 downregulated genes under the prespecified criteria (adjusted P < 0.05 and |log2FC| > 0.5; Figure 1A). To place these alterations within a drug-centric framework, predicted chlorpromazine (CPZ) targets were integrated with GBM differentially expressed genes, yielding 231 intersecting genes, including 94 in the CPZ ∩ Glioma_Up group and 137 in the CPZ ∩ Glioma_Down group (Figure 1B), supporting a multi-target intersection between CPZ-associated genes and GBM dysregulation rather than an isolated single-gene explanation. A high-confidence protein–protein interaction network constructed from these intersecting genes revealed a connected topology with several highly connected cancer-related regulators, such as EGFR and MAPK-family members, indicating convergence on shared signaling backbones (Figure 1C). To further assess the clinical relevance of this intersecting gene set, Lasso-penalized Cox regression was applied to derive a parsimonious prognostic model, with coefficient trajectories and cross-validation supporting stable feature selection (Figures 1D,E). The resulting risk score effectively stratified patients into high- and low-risk groups, with significantly poorer overall survival and disease-specific survival observed in the high-risk group in the TCGA cohort (both log-rank P < 0.001; Figures 1F,G). Associations of the risk score with overall survival, disease-specific survival, and progression-free interval were evaluated as parallel endpoints and summarized in a unified forest-plot format (Figure 1H). Among the genes contributing to this prognostic signature, TTC23 was prioritized for downstream analyses because it remained consistently associated with adverse outcomes and retained relevance within the CPZ–GBM intersecting landscape.

### 3.2 Structure-based docking and conformational assessment of the TTC23–CPZ interaction

**FIGURE 2:**
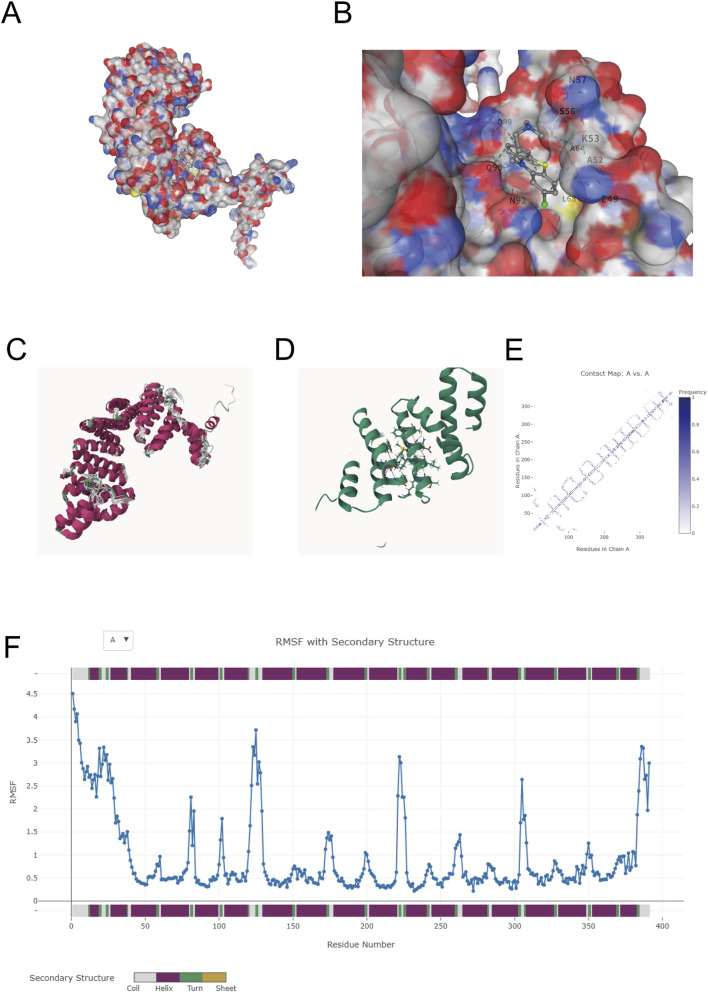
Molecular docking and CABS-flex dynamic simulation verification of TTC23–CPZ interaction. **(A)** Electrostatic potential surface of TTC23 with docked chlorpromazine (CPZ) placed in the predicted pocket (blue, positively charged; red, negatively charged). **(B)** Predicted binding pose highlighting CPZ and key pocket residues; polar contacts and residue labels are shown. **(C)** Superposition of representative TTC23 conformations generated by CABS-flex conformational sampling, illustrating flexibility of the docked complex. **(D)** Cartoon representation of TTC23 with CPZ placed in the pocket. **(E)** Residue–residue contact frequency map derived from the sampled ensemble, indicating contact persistence across conformations. **(F)** Per-residue RMSF profile with secondary-structure annotation; comparatively lower RMSF values in the pocket-lining region are consistent with limited local fluctuations under conformational sampling.

To assess whether TTC23 is structurally capable of interacting with chlorpromazine (CPZ), molecular docking was performed using a three-dimensional TTC23 model. CPZ was predicted to bind within an electrostatically heterogeneous pocket, adopting a pose compatible with both positively and negatively charged surface features (Figure 2A). The top-ranked docking configuration indicated that ligand accommodation is supported by a combination of polar interactions and local packing, with several residues—including E49, A52, K53, S56, N57, L68, and N92—contributing to pocket formation and ligand stabilization (Figure 2B). Visualization of the complex in a cartoon representation further illustrated CPZ placement relative to the surrounding secondary-structure elements (Figure 2D). To complement the static docking results, coarse-grained conformational sampling was performed using CABS-flex. Structural ensemble superposition showed limited conformational dispersion around the predicted binding region (Figure 2C), and contact map analysis revealed persistent intramolecular interaction patterns across the ensemble (Figure 2E). Consistently, RMSF profiling demonstrated relatively low fluctuations for residues lining the ligand-binding pocket compared with more flexible peripheral regions (Figure 2F). Together, these structure-based analyses support the structural plausibility of a putative TTC23–CPZ interaction under conformational sampling, rather than constituting direct experimental evidence of binding.

### 3.3 Global functional landscape of CPZ–GBM intersecting targets

**FIGURE 3:**
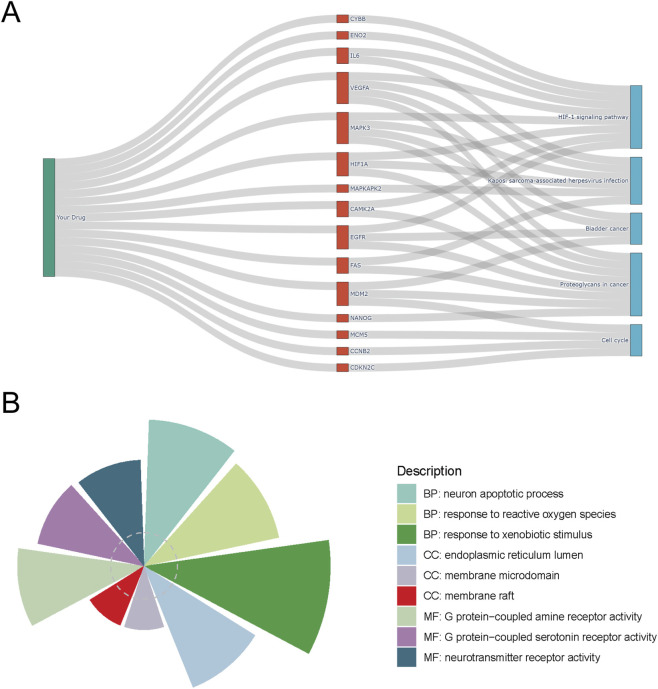
Functional mechanism enrichment of intersecting targets. **(A)** Drug–target–pathway Sankey diagram linking CPZ, representative intersecting targets, and the top enriched KEGG pathways, illustrating multi-target and multi-pathway characteristics. **(B)** GO functional enrichment visualization across BP, CC, and MF categories, summarizing key biological processes, cellular localizations, and molecular functions associated with the intersecting gene set. Abbreviations: GO, Gene Ontology; BP, biological process; CC, cellular component; MF, molecular function; KEGG, Kyoto Encyclopedia of Genes and Genomes.

To contextualize the biological programs represented by the CPZ–GBM intersecting gene set, functional enrichment analyses were performed to characterize the global pharmacological landscape rather than to infer single-gene mechanisms. KEGG pathway analysis highlighted significant enrichment in cancer-relevant pathways, and the drug–target–pathway Sankey diagram illustrated how representative intersecting genes link CPZ to major signaling modules (Figure 3A). Prominently represented pathways included HIF-1 signaling, proteoglycans in cancer, and cell cycle regulation, with key nodes such as HIF1A, VEGFA, EGFR, MAPK3, MDM2, FAS, CCNB2, and CDKN2C bridging hypoxia responses, proliferative control, and stress-related signaling (Figure 3A). Gene Ontology enrichment further supported this pharmacological context, with biological processes enriched for neuron apoptotic process, response to reactive oxygen species, and response to xenobiotic stimulus; cellular components enriched for endoplasmic reticulum lumen and membrane-associated microdomains; and molecular functions emphasizing receptor-related activities, including neurotransmitter and G protein–coupled receptor functions (Figure 3B). Collectively, these enrichment patterns delineate a coordinated functional backdrop in which CPZ-associated targets intersect with GBM dysregulation across hypoxia-related signaling, membrane-associated processes, stress responses, and cell-cycle regulation, providing a systems-level framework for subsequent target-specific investigation.

### 3.4 Aberrant expression and cell cycle–associated dynamics of TTC23 in glioblastoma

**FIGURE 4:**
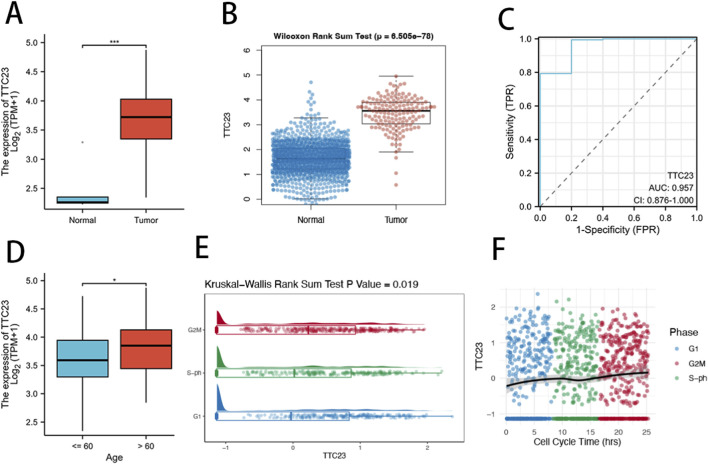
Aberrant expression and cell cycle–associated dynamics of TTC23 in glioblastoma. **(A)** Distribution of TTC23 expression levels across glioblastoma samples in the TCGA-GBM cohort. **(B)** Comparison of TTC23 expression between TCGA-GBM tumors and normal brain tissues from the GTEx project after dataset integration. **(C)** Receiver operating characteristic (ROC) curve evaluating the discriminative performance of TTC23 expression between tumor and normal samples. **(D)** Association between TTC23 expression and patient age in the TCGA-GBM cohort. **(E)** TTC23 expression across distinct cell-cycle phases in single U2OS FUCCI cells from the GSE146773 dataset, with statistical significance assessed using the Kruskal–Wallis test. **(F)** Dynamic expression pattern of TTC23 along the cell-cycle pseudotime trajectory inferred from FUCCI-based single-cell RNA sequencing, illustrating continuous changes associated with cell-cycle progression.

Analysis of the TCGA-GBM cohort revealed that TTC23 is broadly expressed across glioblastoma samples, indicating a tumor-intrinsic expression pattern rather than restriction to a small molecular subset (Figure 4A). Using GTEx normal brain tissues as a reference, TTC23 expression was higher in GBM than in non-tumor brain samples (Figure 4B), with comparisons interpreted cautiously given cohort and platform differences. Consistent with this observation, receiver operating characteristic (ROC) analysis demonstrated that TTC23 expression exhibited strong discriminative performance between tumor and normal samples (Figure 4C), highlighting its potential diagnostic relevance at the transcriptomic level. To assess whether TTC23 expression is confounded by patient demographics, its association with age was examined and showed no dominant age-driven bias across the GBM cohort (Figure 4D), suggesting that TTC23 dysregulation is primarily linked to tumor biology rather than clinical background variables. Beyond static expression differences, analysis of an independent single-cell cell-cycle–resolved dataset revealed that TTC23 expression varies significantly across distinct cell-cycle phases (Figure 4E). Moreover, when single cells were ordered along a cell-cycle pseudotime trajectory, TTC23 displayed a dynamic expression pattern that followed cell-cycle progression rather than remaining constant (Figure 4F). Together, these results indicate that TTC23 expression tracks cell-cycle–associated transcriptional programs, providing a biological rationale to examine proliferation- and survival-related phenotypes in functional assays.

### 3.5 TTC23-associated transcriptional programs highlight cell cycle–related oncogenic activity

**FIGURE 5:**
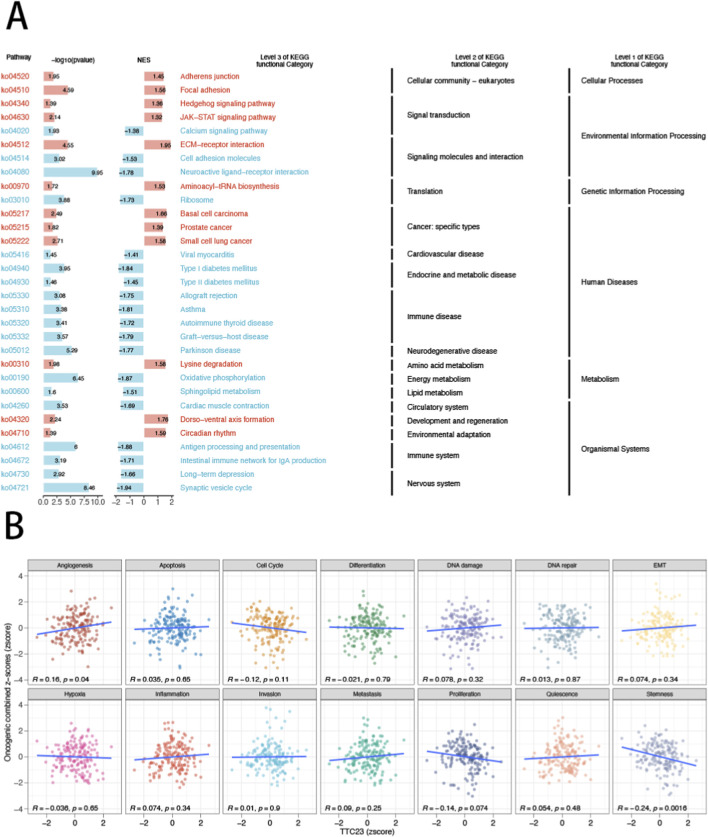
TTC23-associated transcriptional programs and oncogenic state activity in glioblastoma. **(A)** Gene set enrichment analysis (GSEA) comparing tumors with high and low TTC23 expression, highlighting KEGG pathways differentially enriched between the two groups. Samples were stratified using the top and bottom 30% of TTC23 expression. **(B)** Correlation analysis between TTC23 expression and GSVA-derived oncogenic state scores across 14 tumor functional phenotypes, including proliferation, cell cycle, stemness, invasion, and DNA damage–related programs. Each panel displays Pearson correlation coefficients and associated significance values.

To explore the biological programs associated with TTC23 dysregulation in glioblastoma, we performed transcriptome-wide pathway activity analyses by stratifying samples according to TTC23 expression levels. Gene set enrichment analysis revealed that tumors with high TTC23 expression exhibited coordinated activation of multiple cancer-related pathways, particularly those linked to proliferative and cell cycle–associated processes, whereas low TTC23 tumors showed relative depletion of these programs (Figure 5A). Enriched KEGG pathways converged on canonical oncogenic axes, indicating that TTC23 expression is embedded within broader transcriptional states rather than acting as an isolated molecular event. To further resolve functional phenotypes at the pathway-activity level, we quantified oncogenic state scores using a GSVA-based framework across 14 curated tumor functional states. Correlation analysis demonstrated that TTC23 expression was selectively associated with distinct oncogenic programs rather than uniformly correlated across all states (Figure 5B). Notably, TTC23 showed a significant negative association with stemness-related signatures, while displaying context-dependent relationships with proliferation- and cell cycle–linked activities. These results suggest that TTC23-high tumors align with a proliferative transcriptional configuration and altered tumor cell state features, providing a transcriptomic context for the downstream phenotyping analyses.

### 3.6 TTC23 expression defines immune cycle engagement and immunomodulatory architecture in glioblastoma

**FIGURE 6:**
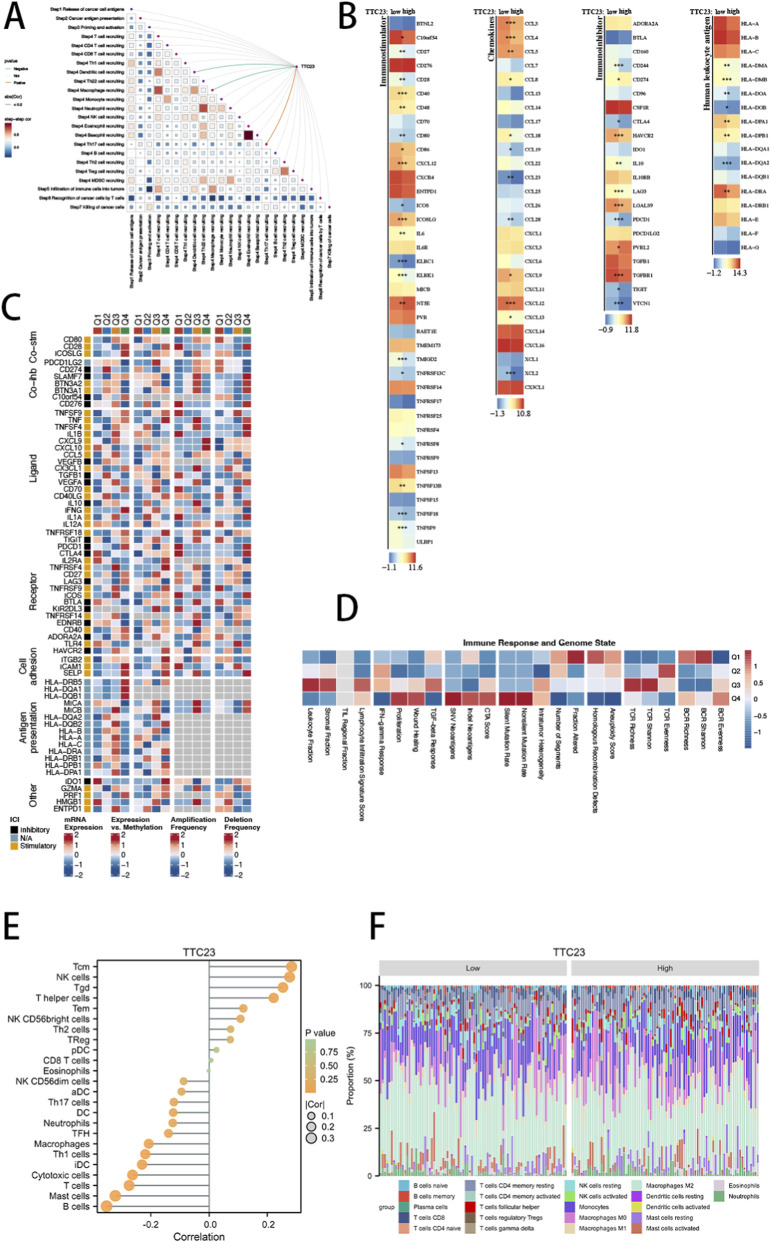
TTC23-associated immune landscape inferred from bulk transcriptomic analyses in glioblastoma. **(A)** Spearman correlation analysis between TTC23 expression and cancer–immunity cycle activity scores derived from the TIP framework, together with the inter-step correlation structure among immune cycle phases. **(B)** Differential expression of immunostimulatory genes, immunoinhibitory genes, chemokines, and HLA family members between TTC23 high- and low-expression groups. **(C)** Integrated analysis of immunomodulator patterns across TTC23 expression quartiles, including mRNA expression levels, expression–methylation associations, and somatic copy number alteration frequencies. **(D)** Heatmap summarizing immune response signatures and genome state features across TTC23 expression quartiles. **(E)** Correlation between TTC23 expression and ssGSEA-estimated immune cell infiltration scores across 24 immune cell populations. **(F)** Distribution of CIBERSORT-inferred immune cell fractions across TTC23 expression groups.

To delineate the immunological context associated with TTC23 expression, we first evaluated its relationship with the cancer–immunity cycle using the TIP framework. TTC23 expression showed selective correlations with specific immune cycle steps rather than uniform association across the entire cascade, with stronger links to immune cell recruitment–related phases and weaker or inverse associations with late effector steps such as tumor cell killing (Figure 6A). These correlations are inference-based and are presented as hypothesis-generating associations rather than experimentally validated immune mechanisms. Importantly, correlation analysis among the seven immune cycle steps revealed preserved inter-step dependencies, indicating that TTC23-associated immune alterations are embedded within an organized immune response architecture rather than reflecting nonspecific immune activation. Consistent with these associations, TTC23 stratification showed coordinated differences in immunostimulatory genes, immunoinhibitory genes, chemokines, and HLA family members (Figure 6B), supporting an immune-linked transcriptional context associated with TTC23 expression.

Beyond transcriptional differences, integrative analyses revealed that TTC23-associated immune patterns extend across multiple regulatory layers. Immunomodulators displayed concordant variation in mRNA expression, DNA methylation–associated regulation, and somatic copy number alteration frequencies across TTC23 expression quartiles (Figure 6C). At the systems level, immune response signatures showed structured associations with tumor-intrinsic genomic states, including proliferative signaling and genome instability–related features (Figure 6D). Finally, immune infiltration analyses using complementary ssGSEA- and CIBERSORT-based frameworks demonstrated that TTC23 expression is linked to coordinated shifts in both innate and adaptive immune cell compartments (Figures 6E,F). Together, these computational analyses outline an immune-associated landscape correlated with TTC23, which can inform future mechanistic and experimental follow-up.

### 3.7 TTC23 mediates the anti-migratory effects of chlorpromazine in glioblastoma cells

**FIGURE 7:**
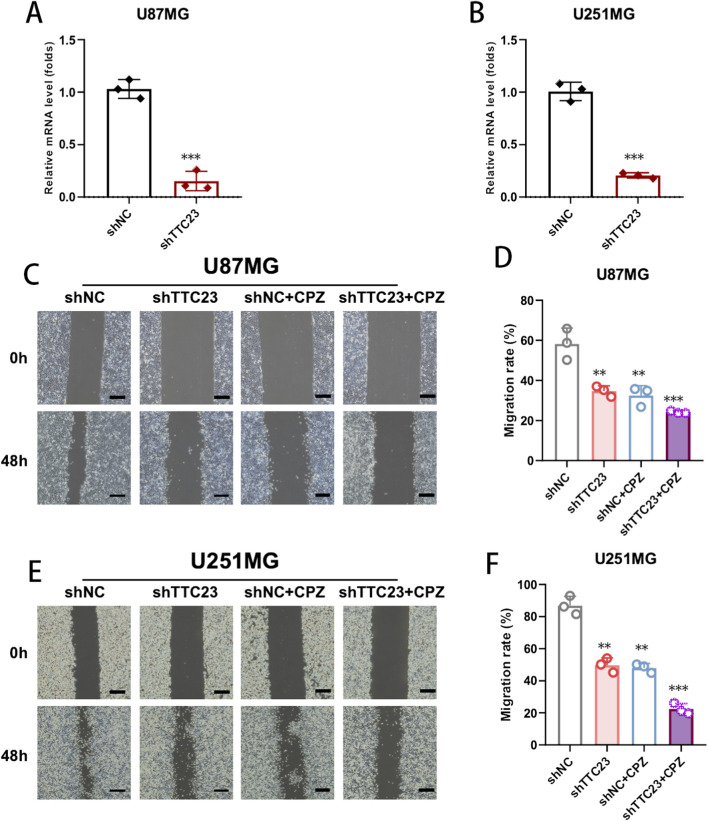
TTC23 knockdown enhances CPZ-associated anti-migratory phenotypes in glioblastoma cells. **(A,B)** Validation of stable TTC23 knockdown in U87MG **(A)** and U251MG **(B)** cells by quantitative PCR. **(C,D)** Wound-healing assay in U87MG cells under the indicated conditions (shNC, shTTC23, shNC + CPZ, and shTTC23 + CPZ), with representative images **(C)** and quantification of migration rates **(D)** at 0 h and 48 h **(E,F)** Wound-healing assay in U251MG cells under the same conditions, with representative images **(E)** and quantification **(F)**. Data are presented as mean ± SD, and statistical tests were performed as described in Methods. **Significance notation: *P < 0.05, **P < 0.01, *P < 0.001.

To functionally validate the role of TTC23 in chlorpromazine (CPZ)–associated anti-glioblastoma phenotypes, stable TTC23 knockdown models were first established in U87MG and U251MG cells using shRNA-mediated gene silencing. Quantitative PCR analysis confirmed a robust and consistent reduction of TTC23 mRNA expression in both cell lines compared with negative control cells, indicating efficient and reproducible knockdown efficiency (Figures 7A,B).

We next examined whether TTC23 contributes to CPZ-mediated suppression of glioblastoma cell migration. Wound-healing assays demonstrated that TTC23 knockdown alone significantly impaired migratory capacity in both U87MG and U251MG cells, supporting a functional role of TTC23 in regulating glioblastoma cell motility (Figures 7C–F). CPZ treatment similarly reduced migration relative to control conditions. Combined TTC23 knockdown and CPZ treatment produced the greatest inhibition of wound closure across both cell models (Figures 7C–F). These results indicate that TTC23 depletion is associated with enhanced anti-migratory effects observed under CPZ exposure, consistent with TTC23 acting as a functional determinant of the CPZ-linked migration phenotype.

### 3.8 TTC23 knockdown potentiates the growth-inhibitory and pro-apoptotic effects of chlorpromazine in glioblastoma cells

**FIGURE 8:**
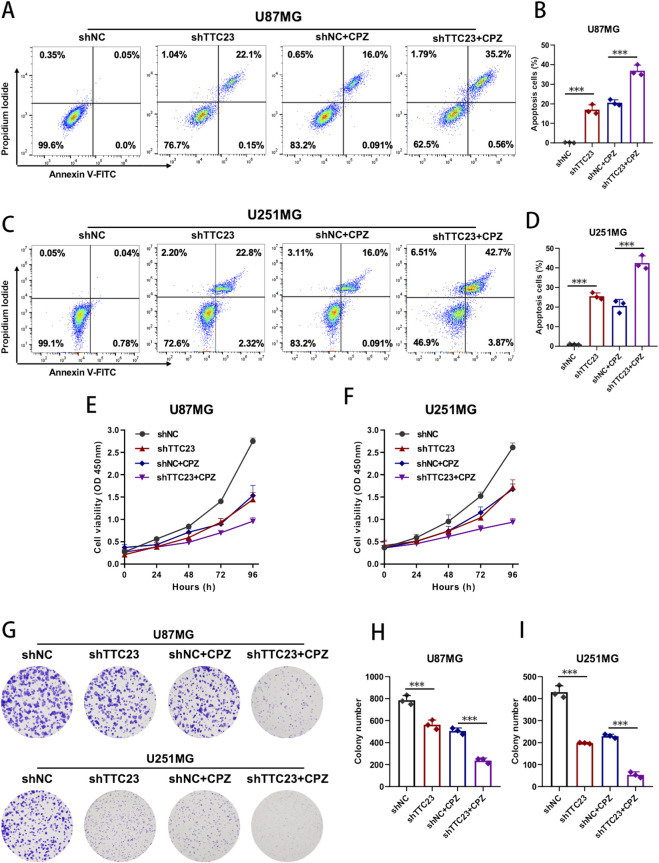
TTC23 knockdown enhances the pro-apoptotic and growth-inhibitory effects of chlorpromazine in glioblastoma cells. **(A,B)** Annexin V/PI flow cytometric analysis of apoptosis in U87MG cells under the indicated conditions (shNC, shTTC23, shNC + CPZ, and shTTC23 + CPZ). Representative dot plots **(A)** and quantitative analysis of apoptotic cell percentages **(B)** are shown. **(C,D)** Annexin V/PI flow cytometric analysis of apoptosis in U251MG cells, with representative plots **(C)** and quantitative results **(D)**. **(E,F)** Cell viability of U87MG **(E)** and U251MG **(F)** cells assessed by CCK-8 assays at the indicated time points under different treatment conditions. **(G)** Representative images of colony formation assays performed in U87MG and U251MG cells. **(H,I)** Quantification of colony numbers in U87MG **(H)** and U251MG **(I)** cells. Data are presented as mean ± SD, and statistical tests were performed as described in Methods. **Significance notation: *P < 0.05, **P < 0.01, *P < 0.001.

To further determine whether TTC23 modulates the anti-tumor activity of chlorpromazine (CPZ) beyond cell migration, we examined its effects on apoptosis induction and tumor cell growth. Flow cytometric analysis using Annexin V/PI staining demonstrated that TTC23 knockdown significantly increased apoptotic cell populations in both U87MG and U251MG cells compared with control conditions (Figures 8A–D). CPZ treatment alone also promoted apoptosis, whereas combined TTC23 knockdown and CPZ exposure resulted in the highest proportion of apoptotic cells across both cell models, indicating a markedly enhanced pro-apoptotic phenotype.

Consistent with these findings, cell viability assays revealed that TTC23 knockdown or CPZ treatment alone moderately suppressed glioblastoma cell growth over time, while the combined intervention produced a more pronounced reduction in cell viability in both U87MG and U251MG cells (Figures 8E,F). Long-term clonogenic assays further confirmed this growth-inhibitory effect, showing that TTC23 knockdown or CPZ treatment individually reduced colony-forming capacity, whereas their combination resulted in the strongest suppression of clonogenic survival (Figures 8G–I). Collectively, these results show that TTC23 depletion is associated with increased apoptotic susceptibility and stronger growth inhibition under CPZ exposure, supporting TTC23 as a functional determinant linked to CPZ-associated anti-tumor phenotypes in GBM cell models.

### 3.9 TTC23 depletion reprograms migration- and immune-related transcriptional programs in glioblastoma cells

**FIGURE 9:**
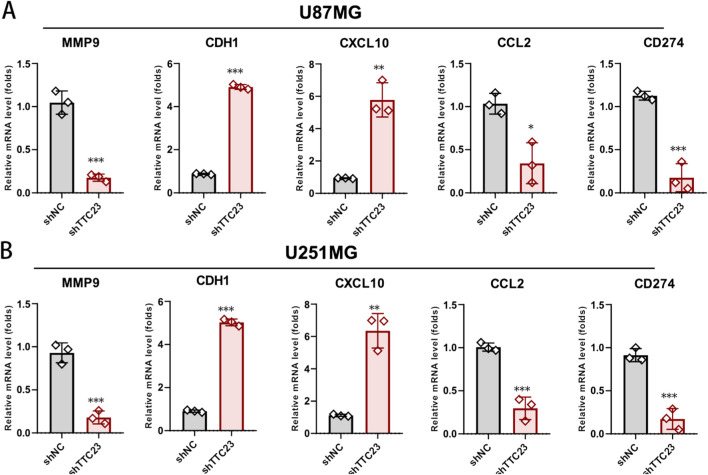
TTC23 knockdown modulates migration-, invasion-, and immune-related gene expression in glioblastoma cells. **(A)** Relative mRNA expression levels of migration-, invasion-, and immune-associated genes (MMP9, CDH1, CXCL10, CCL2, and CD274) in U87MG cells following TTC23 knockdown, as determined by quantitative real-time PCR. **(B)** Relative mRNA expression levels of the same gene panel in U251MG cells after TTC23 knockdown. Data are presented as mean ± SD, and statistical tests were performed as described in Methods. **Significance notation: *P < 0.05, **P < 0.01, *P < 0.001.

To explore the molecular basis underlying the phenotypic changes induced by TTC23 depletion, we examined the transcriptional expression of genes associated with cell migration, invasion, and immune regulation. In both U87MG and U251MG cells, TTC23 knockdown resulted in a significant downregulation of the pro-migratory gene MMP9, accompanied by marked upregulation of the migration-suppressive epithelial marker CDH1 (Figures 9A,B), providing a transcriptional explanation for the impaired migratory capacity observed in functional assays. Notably, TTC23 depletion was also associated with coordinated modulation of immune-related genes, characterized by increased expression of the immune-stimulatory chemokine CXCL10 and concomitant downregulation of the immunosuppressive factors CCL2 and CD274 (PD-L1) (Figures 9A,B). These transcriptional changes are consistent with a shift toward reduced migratory programming and an immune-associated expression pattern under TTC23 depletion, while mechanistic causality will require further dedicated validation.

## 4 Discussion

This study advances a target-centered drug-repurposing rationale for glioblastoma by moving beyond associative bioinformatics toward a coherent, testable model of response (Truong et al., 2022). Rather than claiming a single “magic-bullet” mechanism for a pleiotropic compound, we used a drug–disease intersection framework to prioritize a functionally meaningful node and then experimentally asked whether this node modulates clinically relevant phenotypes. This design choice matters for GBM, where adaptive redundancy and state plasticity frequently undermine pathway-isolated interventions, and where clinically useful targets are those that couple malignant fitness traits (infiltration, survival under stress, and long-term clonogenic potential) to tractable pharmacologic perturbations.

A major implication of our findings is that TTC23 can be positioned as a functional determinant of chlorpromazine (CPZ)-associated anti-tumor phenotypes rather than merely a correlated marker. CPZ has long been recognized as a CNS-active agent with broad molecular interactions; such polypharmacology is an advantage for repurposing in network-adaptive tumors, but it complicates mechanistic clarity (Ryszkiewicz et al., 2023; Kamgar-Dayhoff et al., 2021). In this context, our approach aligns with a pragmatic standard in contemporary translational pharmacology: use systems-level prioritization to reduce polypharmacology into experimentally manageable hypotheses, then validate causality at the phenotype level (Truong et al., 2022). This philosophy is consistent with how many repurposed candidates are now rationalized—by integrating disease transcriptional states, target knowledgebases, and network structure to nominate actionable nodes for validation rather than attempting exhaustive target enumeration.

At the tumor-phenotype level, the most clinically consequential GBM behavior is diffuse infiltration into surrounding brain tissue, which drives surgical non-curability and recurrence (Vaz-Salgado et al., 2023; Wu et al., 2021). The transcriptional shifts we observed upon TTC23 depletion are consistent with an “anti-invasion” directionality, including reduced expression of MMP9 and increased CDH1 (Oishi et al., 2022; Thanh et al., 2024; Noron et al., 2024). MMP9 is widely implicated in ECM remodeling and glioma invasion (Cai et al., 2019), and its regulation is frequently linked to pro-invasive signaling programs in glioma biology (review-level consensus; one commonly cited mechanistic axis is integrin/RTK→MAPK/PI3K→MMP induction) (e.g., general glioma invasion reviews). CDH1 (E-cadherin), while classically discussed in epithelial contexts, is often treated as a marker reflecting adhesion/stability *versus* migratory transition states (Song et al., 2016; Förster et al., 2021); in glioma, adhesion-related state shifts are increasingly conceptualized in terms of transcriptional programs rather than canonical EMT. Framing our observations at this level avoids over-claiming a specific invasion pathway while still providing a biologically coherent explanation for why TTC23 loss suppresses motility.

A second implication concerns the interface between tumor-intrinsic states and immune contexture. Bulk-tumor immune inference approaches—such as ssGSEA-style cell scoring and deconvolution—are now standard for hypothesis generation, but require careful interpretation because they do not prove causality (Yin et al., 2025; Zhao et al., 2023; Zhao et al., 2022; Shen et al., 2022). Nonetheless, they are valuable when they converge with orthogonal evidence. Accordingly, we present the immune-cycle/TIP associations and immunomodulator patterns as hypothesis-generating context rather than direct evidence of immune remodeling. The TTC23-linked transcriptional shifts in CXCL10, CCL2, and CD274 (PD-L1) provide a plausible tumor-intrinsic correlate for these associations, but they do not establish *in vivo* immune effects. CXCL10 is broadly considered a T cell–recruiting chemokine in cancer immune biology, whereas CCL2 is strongly linked to recruitment of monocytes/macrophages and immunosuppressive myeloid states in multiple tumor types; PD-L1 is a central checkpoint ligand shaping immune evasion (Limagne et al., 2022; Pozzi and Satchi-Fainaro, 2024; Li et al., 2024; Vlahovic et al., 2015). Importantly, we do not claim that TTC23 depletion “activates immunity” *in vivo*; instead, we interpret these signals as consistent with a shift toward a less immunosuppressive transcriptional profile that could, in immunocompetent contexts, influence immune-cell trafficking and checkpoint tone. This interpretation is aligned with pan-cancer immune landscape work emphasizing coordinated immune programs and their association with genomic and transcriptional states.

From a pharmacology perspective, the most actionable reading of our data is that TTC23 modulation changes CPZ responsiveness across multiple fitness axes—migration, apoptosis propensity, and long-term clonogenic survival—rather than producing a single isolated readout. This multi-phenotype coherence is important: in GBM, reductions in short-term viability do not necessarily translate into reduced recurrence potential, whereas clonogenic suppression is often used as a stricter proxy for durable growth control. The observation that TTC23 depletion amplifies CPZ-associated apoptosis and suppresses clonogenic survival therefore supports TTC23 as a pharmacologically relevant response determinant. We intentionally avoid labeling this interaction as “synergy” in a formal sense because synergy requires dose–response matrix modeling (e.g., Bliss/Loewe/HSA), but the directionality is consistent with functional potentiation: a tumor-intrinsic state change (TTC23 loss) increases sensitivity to CPZ-induced growth inhibition and cell death.

Several limitations define what should be claimed now *versus* what should be reserved for future work. First, CPZ is polypharmacologic; TTC23 is unlikely to be the sole target mediating all effects, and our conclusion is framed accordingly—TTC23 is a functionally relevant mediator/determinant of CPZ response rather than the only target. Second, structure-informed docking and conformational sampling support structural plausibility but do not demonstrate direct target engagement; the TTC23–CPZ interaction should therefore be interpreted as a testable hypothesis rather than a confirmed binding event. Third, immune implications should be tested in immunocompetent or humanized models to determine whether TTC23-linked chemokine/checkpoint shifts translate into altered immune infiltration or therapy response. Finally, our clinical modeling provides prognostic anchoring, but predictive utility (i.e., identifying patients most likely to benefit from CPZ-based strategies) will require prospective or well-controlled retrospective response-linked datasets.

Despite these constraints, our study provides a compact blueprint for how to translate a repurposed, BBB-penetrant agent into an interpretable target-centered hypothesis in GBM. By anchoring CPZ targets to GBM-specific dysregulation, enforcing convergence with interaction networks, prioritizing candidates with outcome-linked modeling, and validating TTC23 across computationally inferred and *in vitro* functional phenotypes while contextualizing immune and pathway states, we move CPZ repurposing from an observational premise toward a mechanistically grounded, experimentally supported framework. More broadly, this approach illustrates how multi-layer evidence can be assembled to nominate candidate response determinants in a tumor where effective therapy is likely to require coordinated suppression of invasion and survival programs.

## 5 Conclusion

In summary, we present an integrative, target-centered framework that combines transcriptomic profiling, network-based prioritization, and functional phenotyping to refine the rationale for chlorpromazine repurposing in glioblastoma. This framework nominates TTC23 as a biomarker-linked determinant associated with CPZ response and shows that its modulation is accompanied by coordinated changes in multiple malignant fitness traits, including migration, apoptotic susceptibility, and clonogenic survival. Collectively, our findings illustrate how system-level strategies can help translate pleiotropic drugs into interpretable, mechanism-informed and experimentally testable therapeutic hypotheses.

## Data Availability

The datasets analyzed in this study are publicly available. Bulk transcriptomic data were obtained from the Gene Expression Omnibus (GEO) under accession number GSE4290 and from The Cancer Genome Atlas (TCGA-GBM) project. Single-cell RNA-seq data used for cell-cycle–resolved analysis were obtained from GEO under accession number GSE146773. All other data generated during this study are included in the article and its supplementary material or are available from the corresponding author upon reasonable request.
